# A population-based analysis of the global burden of epilepsy across all age groups (1990–2021): utilizing the Global Burden of Disease 2021 data

**DOI:** 10.3389/fneur.2024.1448596

**Published:** 2024-12-12

**Authors:** Ling-zhi Yang, Yi Guo, Zhi-qiang Wang, Chen-qi Zhang

**Affiliations:** ^1^The Department of Neurology, Chengdu BOE Hospital, Chengdu, Sichuan, China; ^2^The Department of Neurology, Sichuan Provincial People’s Hospital, Chengdu, Sichuan, China

**Keywords:** epilepsy, Global Burden of Disease, socioeconomic disparity, time trend, prevalence, incidence, mortality, DALYs

## Abstract

**Objective:**

To investigate the trends in epilepsy prevalence, incidence, mortality, and disability-adjusted life-years (DALYs) in all ages, with risk factors for epilepsy - associated death, from 1990 to 2021.

**Methods:**

Using the standardized Global Burden of Disease (GBD) methodologies, we evaluated the burden of epilepsy in 204 countries and regions from 1990 to 2021, aiming to derive a more precise representation of the health burden posed by epilepsy by considering four distinct types of epidemiological data, namely the prevalence, incidence, mortality, and DALYs. The presented data were meticulously estimated and displayed both as numerical counts and as age-standardized rates per 100,000 persons of the population. All estimates were calculated with 95% uncertainty intervals (UI).

**Finding:**

In 2021, there were 24,220,856 (95% UI: 18,476,943–30,677,995) patients with epilepsy, with an age-standardized prevalence rate (ASPR) of 307.38 per 100,000 persons (95% UI: 234.71–389.02) and an age-standardized incidence rate (ASIR) of 42.821 per 100,000 persons (95% UI: 31.24–53.72).The global age-standardized mortality rate (ASMR) of epilepsy was 1.74 per 100,000 population (95% UI: 1.46–1.92); The age-standardized DALYs rate (ASDR) were 177.85 per 100,000 population (95% UI: 137.66–225.90); 154.25 per 100,000 population for females [114.73–201.76], and 201.29 per 100,000 population for males [157.93–252.74]. All of the ASPR, ASIR, ASMR and ASDR of males were higher than those of females, and the ASIR of epilepsy was the highest in children aged 0–14, at 61.00(95% UI: 39.09–86.21), while the older adult group aged 70+ has the highest ASMR of 5.67(95% UI: 4.76–6.18). From 1990 to 2021, the number of epilepsy-related deaths and DALYs both decreased. However, the ASPR of epilepsy increased by about 6.9% (95% UI: −0.10–0.26), and the ASIR increased by almost 12% (95% UI: 0.05–0.33). The trends in ASPR, ASIR, ASMR and ASDR exhibited notable variations across different regions.

**Conclusion:**

Epilepsy is an increasing global health challenge with rising prevalence and incidence. Results of this cross-sectional study suggest that despite the global decline in deaths and DALYs, Epilepsy remains an important cause of disability and death, especially in low SDI regions. An improved understanding of the epidemiology of epilepsy may potentially have considerable benefits in reducing the global burden of epilepsy, by aiding in policy-making in low-income countries, provide data support for research on epilepsy medications and treatment methods.

## Introduction

Epilepsy is a condition characterized by recurrent epileptic seizures due to abnormal electrical activity in the brain (1, 2). Epilepsy can be classified, depending on its etiology, into several categories, including epilepsy, genetic epilepsy, infectious epilepsy, metabolic epilepsy, structural epilepsy, and immune epilepsy (1).

Patients with epilepsy (PWE) frequently encounter detrimental psychological and societal consequences, such as anxiety, depression, feelings of inferiority, learning difficulties, unemployment, and reduced marriage prospects, all of which contribute to a diminished quality of life (2). In addition, epilepsy confers a mortality rate that is 3–10 times higher than that of the general population, attributable to a spectrum of causes including accidental fatalities, unrelenting epilepticus, and suicide, as well as complications arising from therapeutic interventions for epilepsy (3–5).

Especially in the aftermath of the COVID-19 pandemic, due to psychological stress, isolation measures, and limited access to medical facilities, the prevalence of epilepsy patients has escalated, with approximately 16.4% of those afflicted experiencing an exacerbation in seizure frequency. This trend is particularly prominent among individuals who are overweight or obese, immunocompromised, have an active epilepsy diagnosis, or who are prescribed multiple anti-seizure medications (6). Additionally, during this period, approximately 57.1% of epilepsy patients experienced severe psychological distress (7). These disease dynamics, imposing a significant burden on patients and their families, have emerged as global public health concerns.

These findings indicate that the impact of COVID-19 on PWE is multifaceted, involving increased seizure frequency and exacerbated psychological health issues, underscoring the significance of this study within the current context. Regular reassessment of the Global Burden of Disease (GBD) for patients with Epilepsy, encompassing updated evaluations of risk factors, is paramount in efforts to prevent long-term complications of epilepsy and improve the quality of life for PWE, particularly after the COVID-19 pandemic.

Despite a wealth of research on the epidemiology of epilepsy, a comprehensive understanding of the long-term global trends and disease burden of Epilepsy remains elusive. The COVID-19 pandemic has further complicated the management and treatment of patients with epilepsy. Our study aims to address this gap by conducting an in-depth analysis of the GBD data from 1990 to 2021. Here, we provides a comprehensive assessment of trends in prevalence, incidence, deaths, and related disability-adjusted life years (DALYs) in the epilepsy population, as well as related risk factors. We anticipate that this interpretation of the GBD 2021 estimates for healthcare professionals will catalyze the development of novel preventive, therapeutic strategies as well as risk factors to alleviate the health risks associated with epilepsy.

## Methods

### Data extraction

This cross-sectional study was approved by Chengdu BOE Hospital. The ethical board of Chengdu BOE Hospital granted a waiver of informed consent as the study only involved data analysis and no identifiable personal information.

In our study, the determinations and their 95% uncertainty interval (UI) for prevalence, incidence, mortality and DALYs relating to epilepsy were drawn from the GBD 2021 data (8). The GBD 2021 companion paper details the data input, processing, synthesis, and final model used to predict disease burden (9) (Supplementary material 1). The estimates used in this study are publicly available through the GBD results tool.^1^ According to GBD 2021, the definitions of Epilepsy was from the “Guidelines for Epidemiologic Studies on Epilepsy”: (1) Epilepsy: a condition characterized by recurrent (two or more) epileptic seizures, unprovoked by any immediate identified cause, and (2) “Active” epilepsy: a prevalent case of active epilepsy is defined as a person with epilepsy who has had at least one epileptic seizure in the previous 5 years, regardless of antiepileptic drug (AED) treatment. At the same time, the ICD-10 codes for epilepsy were from G40 (Neuro, epilepsy, total) and G41 (Neuro, epilepsy, status epilepticus) (9, 10).

### Data description

To summarize the age distribution of the burden of epilepsy, patients were divided into 4 groups: 0–14 years, 15–49 years, 50–69 years and older than 70 years.

We collected data regarding the numbers of prevalence, incidence, mortality, and DALYs in epilepsy patients, along with their corresponding age-standardized rates (ASR) at global, regional, and national levels. Direct age-standardization methods are applied, utilizing the global standard population as stipulated by GBD to compute health indicators specific to each age group. The methodology commences with calculating disease rates for each age group, proceeds with weighting these rates based on the standard population, and concludes with the summation to derive an age-standardized disease rate (9). The standardization process is designed to neutralize biases stemming from disparate age structures, thereby augmenting the comparability and scientific validity of the study’s outcomes.

Age-standardized Prevalence Rate (ASPR) measures the proportion of existing disease cases in a population, adjusted for age distribution, allowing for comparable analyses across different demographics and time periods. Age-standardized Incidence Rate (ASIR) reflects the rate at which new cases of a disease emerge within a specific timeframe, standardized by age to account for variations in population age structures.

Age-standardized Mortality Rate (ASMR) indicates the mortality risk associated with a disease, standardized by age to provide a consistent measure of fatality rates across different populations. Lastly, the Age-standardized Disability-Adjusted Life Years Rate (ASDR) combines the impact of premature death and disability, offering a comprehensive assessment of the overall disease burden on a population’s health (9, 10).

Data on the race and ethnicity of the participants are not listed in the GBD database, which does not allocate race and ethnicity for data collection. In addition, we computed the rate change in age-standardized rates and its accompanying 95% confidence interval (CI) to delineate prevalence trends spanning the study period of 1990–2021. Additionally, we also collected data regarding global risk factors that contribute to epilepsy mortality in all ages patients (8). This study followed the Strengthening the Reporting of Observational Studies in Epidemiology (STROBE) reporting guidelines.

### Sociodemographic index

The Sociodemographic Index (SDI) assesses a country or region’s development using fertility, education, and income data. Scores range from 0 to 1, indicating varying degrees of socioeconomic progress. The SDI is linked to disease incidence and mortality rates (1) and the GBD database (8) divides regions into 5 SDI groups. Our study explores the relationship between epilepsy’s burden and socioeconomic development in these regions (11) (Supplementary Table S1).

### Statistics analysis

The prevalence, incidence, mortality, and DALYs were represented as a projection for every 100,000 persons in the populace along with 95% uncertainty interval (UI) according to the GBD algorithm (1) and visualized by locally Weighted Scatterplot Smoothing curves. All procedures for analysis were performed utilizing the statistical computing software, R (Version 4.0.3) and JD_GBDR (V2.22, Jingding Medical Technology Co., Ltd.) was used for the drawing of the figures.

## Results

### Global level

#### General trends

In 2021, the worldwide number of epilepsy cases were 24,220,856 (95% UI: 18,476,943–30,677,995), with an age-standardized prevalence rates (ASPR) of 307.38 per 100,000 persons (95% UI: 234.71–389.02). Rate change in ASPR was 7% that represented an increase of 0.07 per 100,000 persons (95% CI: −0.10–0.26) from 1990 to 2021. However, the global incidence of epilepsy involved 3,272,734 cases (95% UI: 2,403,802–4,125,119), with an age standardized incidence rates (ASIR) of 42.82 per 100,000 persons (95% UI: 31.24–53.72), this rate has increased by 12% (95% CI: −0.05 to −0.33) between 1990 and 2021. The number of deaths remained at 139,851 (95% UI: 116,953–153,370), with an age-standardized mortality rates (ASMR) of 1.74 per 100,000 persons (95% UI: 1.46–1.92), showing a decrease of 0.16 per 100,000 persons (95% CI: −0.23 to −0.09) from 1990 to 2021.The global disability-adjusted life years (DALYs) for epilepsy in 2021 was 13,877,827 (95% UI: 10,732,569–17,619,993), with an age-standardized DALYs rates (ASDR) of 177.85 per 100,000 persons (95% UI: 137.66–225.90), which decreased by 14.5% (95% CI: −0.24 to −0.04) between 1990 and 2021 (Table 1).

**Table 1:** Numbers and age-standardized rates of prevalence, incidence, deaths, and disability-adjusted life-years (DALYs) for idiopathic in review epilepsy in 1990 and 2021 for both sexes and rate change of age standardized rates by Global Burden of disease (GBD).

	Prevalence					Incidence				
	1990		2021		1990–2021	1990		2021		1990–2021
	Numbers (1990)	Age-standardized rates (1990)	Numbers (2021)	Age-standardized rates (2021)	Rate change in age- standardized rates	Numbers (1990)	Age-standardized rates (1990)	Numbers (2021)	Age-standardized rates (2021)	Rate change in age- standardized rates
Global	15,320,820 (11,456,272–19,419,537)	287.46 (215.70–362.96)	24,220,856 (18,476,943–30,677,995)	307.38 (234.71–389.02)	0.069 (−0.097–0.255)	2,121,189 (1,515,389–2,784,665)	38.12 (27.91–49.49)	3,272,734 (2,403,802–4,125,119)	42.82 (31.24–53.72)	0.123 (−0.048–0.326)
Sex										
Male	8,031,753 (6,017,720–10,164,819)	301.58 (227.08–379.46)	12,622,148 (9,672,283–15,882,177)	321.82 (246.90–405.07)	0.067 (−0.102–0.259)	1,127,967 (803,892–1,473,788)	40.17 (29.50–51.85)	1,736,615 (1,279,500–2,176,394)	45.11 (33.08–56.43)	0.123 (−0.049–0.322)
Female	7,289,066 (5,410,501–9,268,891)	274.22 (205.27–346.84)	11,598,708 (8,787,001–14,784,754)	293.40 (223.17–373.00)	0.070 (−0.095–0.254)	993,222 (711,206–1,306,451)	36.12 (26.27–47.11)	1,536,119 (1,128,507–1,948,582)	40.51 (29.41–50.98)	0.122 (−0.048–0.320)
Age group										
0–14 years	5,284,295 (3,817,984–7,189,788)	303.84 (219.53–413.41)	6,095,770 (4,272,871–8,270,631)	302.99 (212.38–411.09)	−0.003 (−0.167–0.216)	971,368 (624,226–1,368,636)	55.85 (35.89–78.70)	1,227,191 (786,363–1,734,488)	61.00 (39.09–86.21)	0.092 (−0.077–0.307)
15–49 years	7,457,747 (5,395,261–9,679,816)	275.15 (199.05–357.13)	11,483,777 (8,348,141–14,751,095)	290.83 (211.42–373.58)	0.057 (−0.106–0.236)	938,503 (625,976-1,301,603)	34.63 (23.10–48.02)	1,483,536 (1,037,646–2,001,933)	37.57 (26.28–50.70)	0.085 (−0.080–0.267)
50–69 years	1,752,790 (1,300,176–2,319,021)	257.01 (190.64–340.04)	4,079,966 (2,796,257–5,617,968)	284.01 (194.65–391.08)	0.105 (−0.079–0.293)	156,265 (106,802–220,983)	22.91 (15.66–32.40)	378,547 (257,675–555,659)	26.35 (17.94–38.68)	0.150 (−0.049–0.360)
70+ years	825,987 (607,417–1,087,207)	408.89 (300.69–538.20)	2,561,344 (1,876,377–3,361,007)	518.11 (379.55–679.86)	0.267 (0.049–0.473)	55,052 (36,513–73,693)	27.25 (18.08–36.48)	183,460 (121,202–248,808)	37.11 (24.52–50.33)	0.362 (0.093–0.621)
SDI										
High SDI	2,874,983 (2,077,734–3,717,415)	323.52 (233.96–417.19)	4,192,882 (2,841,951–5,535,550)	357.35 (242.69–470.32)	0.105(−0.116–0.313)	390,075 (268,615–511,963)	46.96 (32.25–62.14)	514,418 (342,283–684,799)	52.05 (34.37–70.31)	0.108 (−0.116–0.321)
High middle SDI	2,771,601 (2,019,357–3,491,164)	262.35 (192.06–332.36)	3,488,443 (2,452,172–4,627,887)	266.45 (185.63–353.86)	0.016 (−0.203–0.243)	350,385 (246,753–454,060)	33.40 (23.45–43.71)	426,210 (289,243–567,787)	37.05 (24.87–50.28)	0.109 (−0.124–0.366)
Middle SDI	4,828,175 (3,502,836–6,175,502)	279.06 (204.04–356.33)	7,330,502 (5,383,584–9,313,155)	303.03 (223.38–384.61)	0.086 (−0.133–0.378)	665,330 (463,426–896,157)	36.37 (25.80–48.39)	964,574 (693,889–1,233,906)	41.74 (29.83–53.30)	0.148 (−0.080–0.451)
Low middle SDI	3,262,716 (2,121,347–4,528,532)	277.87 (182.03–385.60)	5,744,184 (4,244,875–7,423,713)	301.71 (224.05–388.10)	0.086 (−0.186–0.507)	464,657 (292,495–659,137)	36.09 (23.48–49.65)	798,512 (580,952–1,032,876)	40.89 (30.19–52.23)	0.133 (−0.155–0.574)
Low SDI	1,566,135 (854,321–2,318,952)	308.54 (169.15–454.95)	3,443,197 (2,342,908–4,625,232)	308.01 (214.68–412.50)	−0.002 (−0.225–0.404)	248,591 (140,511–368,640)	42.59 (24.16–63.41)	566,338 (370,984–776,083)	45.36 (30.11–60.72)	0.065 (−0.167–0.495)
Regions										
Andean Latin America	218,441 (106,344–330,389)	588.34 (291.13–894.41)	371,993 (204,833–543,762)	561.91 (309.61–821.66)	−0.045 (−0.537–1.055)	28,862 (13,753–43,604)	70.31 (33.90–105.82)	47,503 (25,464–68,780)	71.71 (38.67–103.62)	0.020 (−0.499–1.206)
Australasia	67,417 (25,764–101,759)	328.06 (125.70–497.34)	101,649 (38,961–157,095)	316.15 (123.34–491.00)	−0.036 (−0.621–1.414)	9,706 (3,718–14,839)	49.72 (18.96–76.11)	13,634 (5,442–21,149)	48.85 (19.76–75.95)	−0.017 (−0.602–1.457)
Caribbean	136,011 (87,635–186,348)	383.97 (250.71–524.18)	176,980 (113,531–241,273)	371.13 (236.31–507.39)	−0.033 (−0.372–0.472)	19,528 (12,175–27,001)	52.57 (33.26–72.62)	24,075 (15,128–33,912)	52.51 (32.89–73.75)	−0.001 (−0.356–0.542)
Central Asia	291,666 (185,928–396,769)	417.28 (265.17–564.13)	426,437 (248,479–588,517)	449.70 (262.36–619.74)	0.078 (−0.328–0.707)	33,720 (20,903–48,052)	44.40 (27.70–62.05)	45,659 (26,279–63,772)	47.45 (27.54–66.25)	0.069 (−0.339–0.699)
Central Europe	486,475 (335,292–647,315)	384.12 (263.14–508.29)	469,461 (319,030–621,936)	387.92 (266.44–516.68)	0.010 (−0.245–0.369)	47,505 (31,841–64,464)	40.55 (27.07–55.46)	40,010 (26,684–53,769)	41.92 (27.96–58.95)	0.034 (−0.237–0.373)
Central Latin America	974,473 (680,562–1,305,078)	592.23 (416.26–777.84)	1,366,550 (977,005–1,818,934)	537.38 (384.22–714.33)	−0.093 (−0.333–0.252)	131,059 (87,882–180,479)	72.38 (49.82–97.30)	173,731 (121,135–229,075)	69.70 (48.39–92.19)	−0.037 (−0.287–0.310)
Central Sub–Saharan Africa	224,228 (83,901–382,086)	413.62 (154.59–700.47)	525,248 (234,775–846,638)	394.29 (177.84–625.77)	−0.047 (−0.592–1.539)	35,246 (12,865–62,096)	54.87 (20.05–95.23)	85,155 (34,158–138,874)	55.60 (22.40–88.94)	0.013 (−0.585–1.858)
East Asia	2,290,355 (1,608,136–3,023,614)	192.40 (135.44–256.00)	3,220,037 (2,284,534–4,176,094)	216.08 (149.91–279.23)	0.123 (−0.188–0.567)	274,895 (185,205–376,908)	22.55 (15.41–31.19)	372,849 (259,684–491,425)	28.18 (19.18–37.38)	0.249 (−0.096–0.725)
Eastern Europe	613,743 (432,489–798,547)	268.40 (188.06–351.54)	488,971 (332,912–657,815)	226.46 (152.25–303.93)	−0.156 (−0.369–0.104)	78,797 (55,522–104,606)	37.21 (26.12–49.80)	59,543 (39,815–81,362)	33.15 (22.04–45.28)	−0.109 (−0.320–0.154)
Eastern Sub-Saharan Africa	661,887 (372,679–983,798)	345.83 (197.48–516.91)	1,507,049 (1,014,884–2,022,189)	355.15 (242.05–477.80)	0.027 (−0.253–0.577)	113,424 (62,875–167,901)	50.54 (28.52–74.33)	258,133 (167,483–358,826)	54.02 (35.99–73.18)	0.069 (−0.217–0.638)
High–income Asia Pacific	450,017 (286,634–606,854)	262.53 (166.50–354.90)	563,219 (344,728–766,675)	276.68 (168.61–384.37)	0.054 (−0.285–0.492)	67,592 (42,835–91,927)	41.83 (26.26–57.00)	71,299 (44,656–98,720)	45.04 (27.44–62.64)	0.077 (−0.260–0.497)
High–income North America	841,620 (565,916–1,127,799)	296.98 (199.02–400.78)	1,316,091 (864,692–1,779,808)	334.75 (218.92–460.69)	0.127 (−0.131–0.370)	96,535 (64,181–130,221)	36.53 (24.65–49.71)	143,834 (90,670–199,908)	42.17 (26.95–59.59)	0.155 (−0.107–0.388)
North Africa and Middle East	1,137,471 (738,842–1,583,544)	311.91 (205.43–423.98)	1,940,596 (1,322,545–2,650,490)	316.21 (217.48–430.74)	0.014 (−0.307–0.445)	167,812 (105,398–237,700)	42.98 (27.55–59.61)	298,247 (202,461–422,106)	47.37 (32.28–66.75)	0.102 (−0.238–0.567)
Oceania	16,311 (7,560–26,764)	256.30 (119.74–416.27)	33,850 (14,242–54,211)	248.81 (111.19–390.48)	−0.029 (−0.577–1.206)	2,180 (1,007–3,732)	30.13 (13.86–50.39)	4,420 (1,714–7,277)	29.71 (11.89–48.40)	−0.014 (−0.576–1.324)
South Asia	2,706,515 (1,714,523–3,856,614)	247.67 (158.83–349.79)	4,707,517 (3,434,380–6,020,088)	259.69 (190.70–329.90)	0.049 (−0.250–0.577)	362,107 (223,755–517,303)	30.80 (19.05–43.33)	602,050 (428,444–784,538)	33.10 (23.43–42.49)	0.075 (−0.233–0.578)
Southeast Asia	1,036,484 (723,418–1,429,445)	220.42 (156.61–304.46)	1,786,714 (1,252,879–2,367,345)	263.00 (186.26–349.70)	0.193 (−0.128–0.649)	162,702 (111,437–229,023)	31.97 (22.49–44.24)	248,332 (170,940–336,053)	37.14 (25.44–50.84)	0.162 (−0.147–0.582)
Southern Latin America	157,188 (76,498–233,218)	316.70 (154.41–468.35)	232,854 (112,595–354,907)	339.74 (161.05–515.58)	0.073 (−0.449–1.205)	23,702 (10,904–36,220)	47.08 (21.61–71.81)	32,871 (15,909–49,491)	51.77 (25.01–78.41)	0.100 (−0.455–1.247)
Southern Sub–Saharan Africa	210,456 (136,597–285,095)	414.11 (273.55–553.03)	316,112 (206,632–431,267)	403.70 (271.20–545.31)	−0.025 (−0.330–0.471)	31,773(20,749–44,051)	55.81 (36.38–76.44)	46,002 (29,386–63,986)	56.65 (36.76–78.63)	0.015 (−0.307–0.511)
Tropical Latin America	705,629 (462,654–964,207)	463.34 (302.27–624.74)	895,035 (601,783–1,212,580)	389.51 (262.36–529.02)	−0.159 (−0.398–0.265)	99,702 (62,033–138,529)	61.07 (38.36–84.02)	121,093 (81,539–161,143)	55.24 (37.05–73.69)	−0.096 (−0.350–0.345)
Western Europe	1,386,527 (928,587–1,819,268)	352.02 (236.35–460.49)	1,858,838 (1,138,740–2,480,045)	381.08 (238.05–502.46)	0.083 (−0.213–0.443)	213,652 (141,219–284,517)	60.18 (39.83–81.61)	260,298 (164,172–344,965)	65.28 (41.26–89.06)	0.085 (−0.212–0.446)
Western Sub–Saharan Africa	707,905 (447,928–1,000,001)	369.04 (238.37–525.16)	1,915,656 (1,369,003–2,513,697)	404.55 (293.88–526.12)	0.096 (−0.151–0.521)	120,692 (75,211–173,256)	53.45 (33.48–75.54)	323,994 (220,101–431,752)	59.26 (42.17–77.35)	0.109 (−0.139–0.515)

	Mortality					DALYs				
	1990		2021		1990–2021	1990		2021		1990–2021
	Numbers (1990)	Age-standardized rates (1990)	Numbers (2021)	Age-standardized rates (2021)	Rate change in age-standardized rates	Numbers (1990)	Age-standardized rates (1990)	Numbers (2021)	Age-standardized rates (2021)	Rate change in age-standardized rates
Global	104,356 (85,059–113,885)	2.07 (1.70–2.25)	139,851 (116,953–153,370)	1.74 (1.46–1.92)	−0.158 (−0.228–−0.088)	11,378,954 (8,902,710–14,231,577)	208.08 (163.21–260.35)	13,877,827 (10,732,569–17,619,993)	177.85 (137.66–225.90)	−0.145 (−0.242–−0.042)
Sex										
Male	62,706 (50,779–70,385)	2.57 (2.12–2.88)	83,871 (69,903–94,435)	2.14 (1.78–2.41)	−0.167 (−0.238–−0.093)	6,394,581 (5,094,694–7,800,351)	233.74 (187.17–284.31)	7,891,536 (6,182,334–9,856,272)	201.29 (157.93–252.74)	−0.139 (−0.232–−0.037)
Female	41,651 (28,232–48,921)	1.62 (1.10–1.89)	55,980 (39,449–63,074)	1.36 (0.96–1.54)	−0.159 (−0.283–−0.037)	4,984,372 (3,595,754–6,364,531)	182.90 (132.52–234.56)	5,986,292 (4,439,424–7,794,993)	154.25 (114.73–201.76)	−0.157 (−0.277–−0.022)
Age group										
0–14 years	25,768 (17,567–30,914)	1.48 (1.01–1.78)	18,171 (13,891–21,418)	0.90 (0.69–1.07)	−0.390 (−0.510–−0.222)	4,188,140 (3,112,385–5,405,105)	240.82 (178.96–310.79)	3,564,497 (2,700,944–4,753,410)	177.17 (134.25–236.27)	−0.264 (−0.377–−0.123)
15–49 years	52,504 (44,526–57,963)	1.94 (1.64–2.14)	64,693 (54,671–72,386)	1.64 (1.39–1.83)	−0.154 (−0.232–−0.079)	5,745,062 (4,522,196–7,071,108)	211.96 (166.84–260.88)	7,308,527 (5,688,165–9,081,754)	185.09 (144.06–230.00)	−0.127 (−0.215–−0.037)
50–69 years	16,197 (13,560–17,826)	2.38 (1.99–2.61)	28,97 0(23,692–31,615)	2.02 (1.65–2.20)	−0.151 (−0.224–−0.069)	1,061,997 (827,667–1,369,726)	155.72 (121.36–200.84)	2,002,068 (1,504,917–2,729,083)	139.37 (104.76–189.98)	−0.105 (−0.227–0.008)
70+ years	9,888 (8,293–10,982)	4.90 (4.11–5.44)	28,017 (23,538–30,533)	5.67 (4.76–6.18)	0.158 (0.047–0.276)	383,754 (284,072–501,385)	189.97 (140.63–248.20)	1,002,735 (736,854–1,374,469)	202.83 (149.05–278.03)	0.068 (−0.083–0.229)
SDI										
High SDI	9,610 (9,347–9,968)	1.00 (0.98–1.05)	17,447 (15,721–18,584)	1.08 (1.00–1.14)	0.075 (−0.014–0.133)	1,177,932 (844,227–1,647,445)	133.21 (95.31–186.37)	1,466,497 (992,406–2,150,301)	125.90 (84.94–188.08)	−0.055 (−0.202–0.102)
High middle SDI	15,683 (14,231–17,683)	1.50 (1.36–1.69)	14,051 (12,417–15,374)	0.93 (0.83–1.02)	−0.377 (−0.447–−0.310)	1,783,634 (1,402,274–2,238,661)	168.31 (132.36–211.39)	1,443,736 (1,021,983–2,021,723)	112.64 (79.87–157.06)	−0.331 (−0.454–−0.175)
Middle SDI	29,033 (24,674–32,113)	1.83 (1.57–2.03)	28,709 (24,686–31,674)	1.15 (0.99–1.27)	−0.371 (−0.424–−0.314)	3,543,162 (2,789,455–4,471,812)	199.16 (158.06–250.27)	3,519,220 (2,606,430–4,601,491)	145.96 (108.41–192.62)	−0.267 (−0.390–−0.126)
Low middle SDI	29,745 (20,270–34,343)	3.08 (2.13–3.54)	42,612 (32,664–48,021)	2.51 (1.91–2.83)	−0.185 (−0.297–−0.061)	3,061,150 (2,192,306–3,945,334)	256.25 (185.08–329.45)	4,116,015 (3,207,700–5,233,554)	215.13 (168.21–270.45)	−0.160 (−0.295–0.034)
Low SDI	20,198 (16,059–23,512)	5.69 (4.63–6.71)	36,906 (29,986–43,935)	4.64 (3.76–5.43)	−0.184 (−0.281–−0.075)	1,802,907 (1,362,683–2,320,104)	366.95 (280.89–467.39)	3,320,413 (2,619,841–4,187,258)	308.08 (246.46–378.61)	−0.160 (−0.276–−0.020)
Regions										
Andean Latin America	869 (714–976)	2.54 (2.13–2.86)	912 (755–1,093)	1.40 (1.16–1.68)	−0.451 (−0.551–−0.340)	135,571 (83,412–195,377)	354.34 (216.39–509.86)	154,769 (96,445–230,916)	232.53 (144.45–347.51)	−0.344 (−0.629–0.154)
Australasia	272 (260–286)	1.27 (1.22–1.34)	361 (334–386)	0.93 (0.88–1.00)	−0.265 (−0.317–−0.209)	29,544 (18,212–47,650)	143.84 (88.31–232.56)	35,307 (19,319–62,512)	111.57 (60.77–199.67)	−0.224 (−0.589–0.480)
Caribbean	817 (723–952)	2.53 (2.28–2.91)	1,012 (840–1,221)	2.04 (1.69–2.46)	−0.194 (−0.320–−0.060)	92,044 (67,379–122,097)	258.44 (191.91–341.70)	104,675 (75,798–143,105)	220.70 (159.64–303.92)	−0.146 (−0.359–0.099)
Central Asia	1,577 (1,497–1,697)	2.32 (2.21–2.51)	2,503 (2,203–2,820)	2.59 (2.29–2.92)	0.116 (−0.023–0.269)	197,995 (148,924–256,252)	275.96 (206.67–358.71)	270,937 (207,394–353,474)	280.70 (214.17–367.36)	0.017 (−0.229–0.329)
Central Europe	2,059 (1,986–2,218)	1.57 (1.52–1.69)	2,862 (2,346–3,210)	1.82 (1.49–2.04)	0.157 (−0.071–0.316)	245,248 (180,839–320,805)	195.26 (144.17–252.38)	216,270 (159,252–295,130)	174.56 (127.55–239.83)	−0.106 (−0.304–0.123)
Central Latin America	3,534 (3,427–3,633)	2.44 (2.37–2.50)	4,826 (4,221–5,499)	1.89 (1.65–2.16)	−0.223 (−0.321–−0.117)	567,908 (426,846–749,071)	336.06 (253.03–445.57)	636,587 (455,770–857,865)	250.72 (179.25–337.21)	−0.254 (−0.414–−0.070)
Central Sub–Saharan Africa	1,877 (1,447–2,471)	4.51 (3.54–5.99)	4,153 (3,086–5,731)	3.91 (2.88–5.41)	−0.134 (−0.329–0.105)	207,615 (135,693–295,819)	385.37 (254.12–552.54)	441,289 (287,627–628,281)	334.52 (220.92–477.24)	−0.132 (−0.423–0.312)
East Asia	21,914 (18,681–26,468)	1.84 (1.58–2.22)	12,444 (10,330–15,388)	0.82 (0.69–1.01)	−0.556 (−0.629–−0.460)	2,196,274 (1,770,196–2,725,397)	177.66 (143.49–219.55)	1,435,032 (1,023,185–1,955,288)	102.03 (74.13–138.71)	−0.426 (−0.561–−0.253)
Eastern Europe	2,440 (2,388–2,491)	1.04 (1.02–1.07)	1,570 (1,385–1,743)	0.63 (0.55–0.70)	−0.401 (−0.467–−0.337)	304,675 (229,228–411,467)	134.89 (102.22–181.08)	185,111 (125,736–264,284)	87.13 (58.08–124.66)	−0.354 (−0.498–−0.189)
Eastern Sub-Saharan Africa	10,948 (9,067–12,727)	9.40 (7.79–11.05)	20,643 (17,587–24,914)	7.49 (6.44–8.92)	−0.204 (−0.311–−0.075)	880,336 (691,613–1,108,008)	494.35 (394.15–609.04)	1,697,817 (1,361,673–2,111,472)	423.53 (345.14–519.67)	−0.143 (−0.272–0.019)
High–income Asia Pacific	1,093 (1,015–1,321)	0.62 (0.58–0.75)	2,303 (2,011–2,517)	0.71 (0.66–0.75)	0.134 (−0.062–0.251)	178,485 (122,086–257,556)	105.60 (71.56–152.91)	181,526 (116,478–288,640)	92.92 (59.13–144.30)	−0.120 (−0.385–0.238)
High-income North America	2,020 (1,960–2,069)	0.65 (0.63–0.66)	3,822 (3,582–3,979)	0.79 (0.75–0.81)	0.214 (0.173–0.248)	291,862 (193,788–427,459)	102.52 (67.61–152.19)	423,104 (271,625–643,343)	109.43 (69.25–167.98)	0.067 (−0.138–0.319)
North Africa and Middle East	6,898 (5,560–8,124)	2.25 (1.91–2.62)	7,374 (5,915–8,547)	1.32 (1.08–1.52)	−0.412 (−0.486–−0.330)	869,077 (654,676–1,120,568)	232.93 (177.79–298.87)	982,282 (700,420–1,361,899)	158.62 (113.88–219.76)	−0.319 (−0.471–−0.115)
Oceania	91 (61–131)	1.57 (1.05–2.30)	229 (165–329)	1.73 (1.25–2.48)	0.104 (−0.130–0.419)	11,921 (7,305–17,517)	182.50 (110.26–268.49)	26,577 (16,444–39,292)	187.92 (116.00–276.38)	0.030 (−0.358–0.639)
South Asia	32,077 (20,640–37,634)	3.56 (2.30–4.20)	41,859 (31,308–47,631)	2.58 (1.87–2.94)	−0.277 (−0.389–−0.122)	2,982,571 (2,059,675–3,805,678)	266.28 (184.04–341.67)	3,612,472 (2,845,810–4,472,974)	199.21 (156.41–246.16)	−0.252 (−0.385–−0.054)
Southeast Asia	2,707 (1,933–3,101)	0.76 (0.56–0.85)	3,647 (2,597–4,322)	0.56 (0.40–0.66)	−0.262 (−0.355–−0.154)	544,766 (370,902–778,561)	116.45 (79.83–163.40)	755,399 (512,525–1,060,685)	110.49 (75.07–155.65)	−0.051 (−0.286–0.242)
Southern Latin America	558 (535–583)	1.16 (1.11–1.21)	761 (714–811)	1.01 (0.95–1.08)	−0.129 (−0.192–−0.056)	79,648 (47,913–120,187)	160.53 (96.86–242.45)	93,165 (55,654–147,779)	136.67 (80.72–216.41)	−0.149 (−0.490–0.460)
Southern Sub-Saharan Africa	1,159 (968–1,383)	2.69 (2.23–3.26)	1,920 (1,668–2,371)	2.54 (2.21–3.10)	−0.053 (−0.221–0.161)	142,907 (108,328–189,290)	279.01 (211.33–367.09)	206,134 (155,806–263,097)	257.17 (195.20–330.47)	−0.078 (−0.259–0.183)
Tropical Latin America	1,781 (1,713–1,853)	1.31 (1.26–1.36)	3,795 (3,622–3,937)	1.55 (1.47–1.61)	0.183 (0.118–0.242)	352,884 (247,162–500,208)	229.84 (162.51–321.72)	422,868 (301,544–584,924)	183.07 (130.23–255.98)	−0.204 (−0.397–0.071)
Western Europe	5,085 (4,940–5,197)	1.12 (1.10–1.14)	10,905 (9,548–11,837)	1.41 (1.26–1.52)	0.259 (0.131–0.346)	545,958 (389,498–795,770)	139.60 (98.93–202.50)	668,962 (460,088–990,189)	135.00 (91.09–203.98)	−0.033 (−0.256–0.219)
Western Sub–Saharan Africa	4,580 (3,552–5,928)	3.52 (2.69–4.58)	11,950 (7,788–14,810)	3.64 (2.42–4.47)	0.033 (−0.317–0.432)	521,664 (366,098–722,216)	295.17 (212.27–398.72)	1,327,545 (945,495–1,756,096)	299.72 (215.99–386.81)	0.015 (−0.223–0.281)

#### Overall age and sex distribution

**Figure 1:**
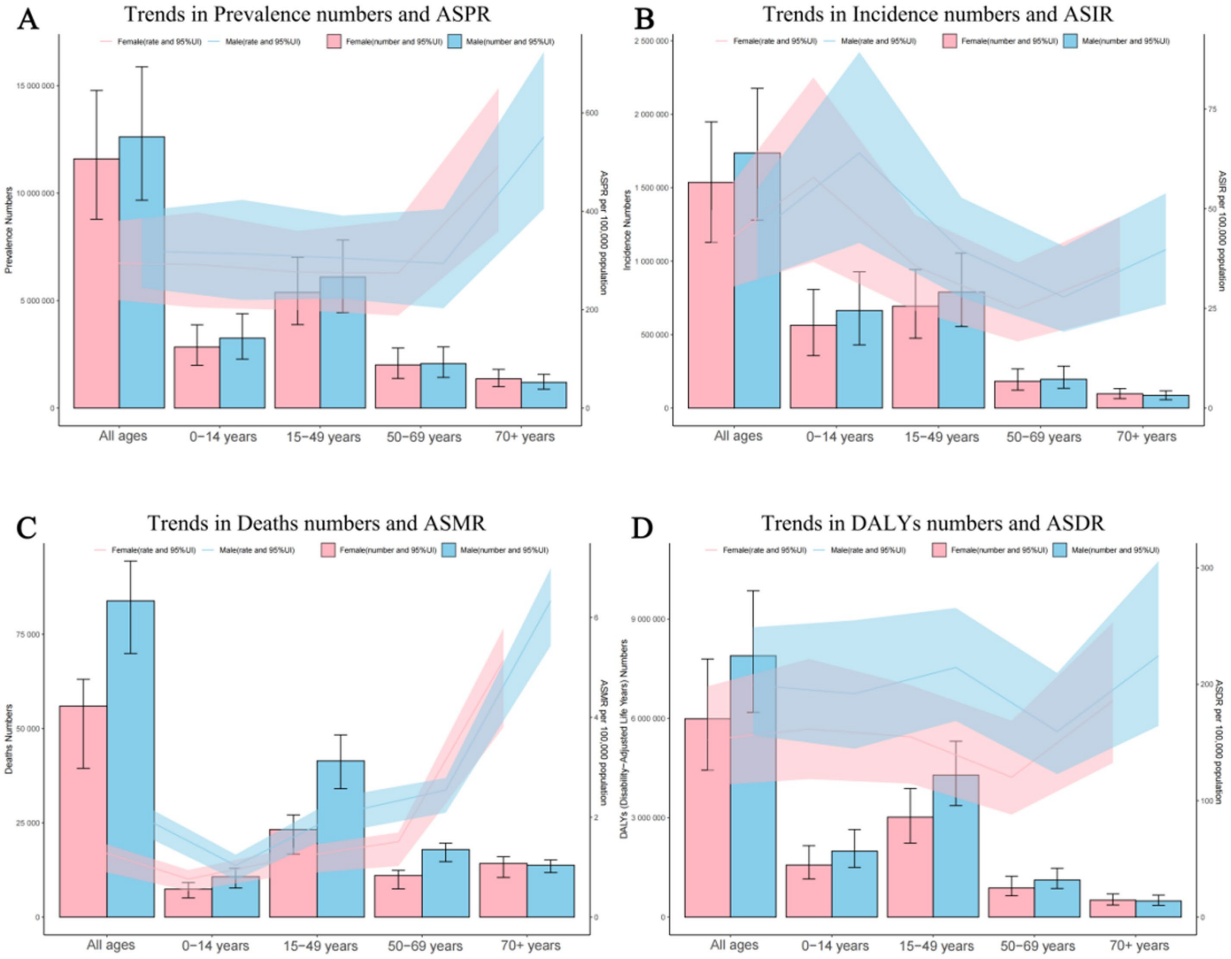
Age and sex trends in idiopathic epilepsy prevalence, incidence, deaths, and disability-adjusted life-years (DALYs) from 1990 to 2021. (A) Trends in prevalence numbers and ASPR. (B) Trends in incidence numbers and ASIR. (C) Trends in deaths numbers and ASMR. (D) Trends in DALYs numbers and ASDR. ASPR, Age-standardized prevalence rates; ASIR, Age-standardized incidence rates; ASMR, Age-standardized mortality rates; ASDR, Age-standardized DALYs rates.

In 2021, the prevalence number of epilepsy patients are generally higher in males than in females, except that the number of female patients is higher than that of male patients in the age group of 70 and above. The highest global prevalence cases of epilepsy was observed in the age group of 15 to 49, with about 11,483,777 patients, accounting for 47.41% of the total age group, including 6,099,124 males and 5,384,652 females. Across all five age brackets, the global ASPR of epilepsy was higher among males than females, with the highest ASPR observed in individuals aged 70 and above (Figure 1A; Supplementary Table 2A). As for the incidence numbers and rates of new epilepsy cases were all generally higher in males compared to females, the incidence rate among children aged 0–14(61.00; 95% UI: 39.09–86.21) is significantly higher than that in other age groups (Figure 1B; Supplementary Table 2B). The mortality rates were highest in the above-70 age group, with the male mortality rates contribution per 100,000 was greater than that of females (6.33 vs. 5.15) (Figure 1C; Supplementary Table 2C).The DALYs rates analysis revealed a trend similar to that of the prevalence, the age group of 70 and above has the highest age-standardized DALYs rates, with around 224.45 per 100,000 persons in males, followed by the 15–49 and 0–14 age groups, Notably, within these four age categories, the contribution of male DALYs rates per 100,000 was superior to that observed among females (Figure 1D; Supplementary Table 2D).

### SDI regional level

#### Prevalence trends

**Figure 2:**
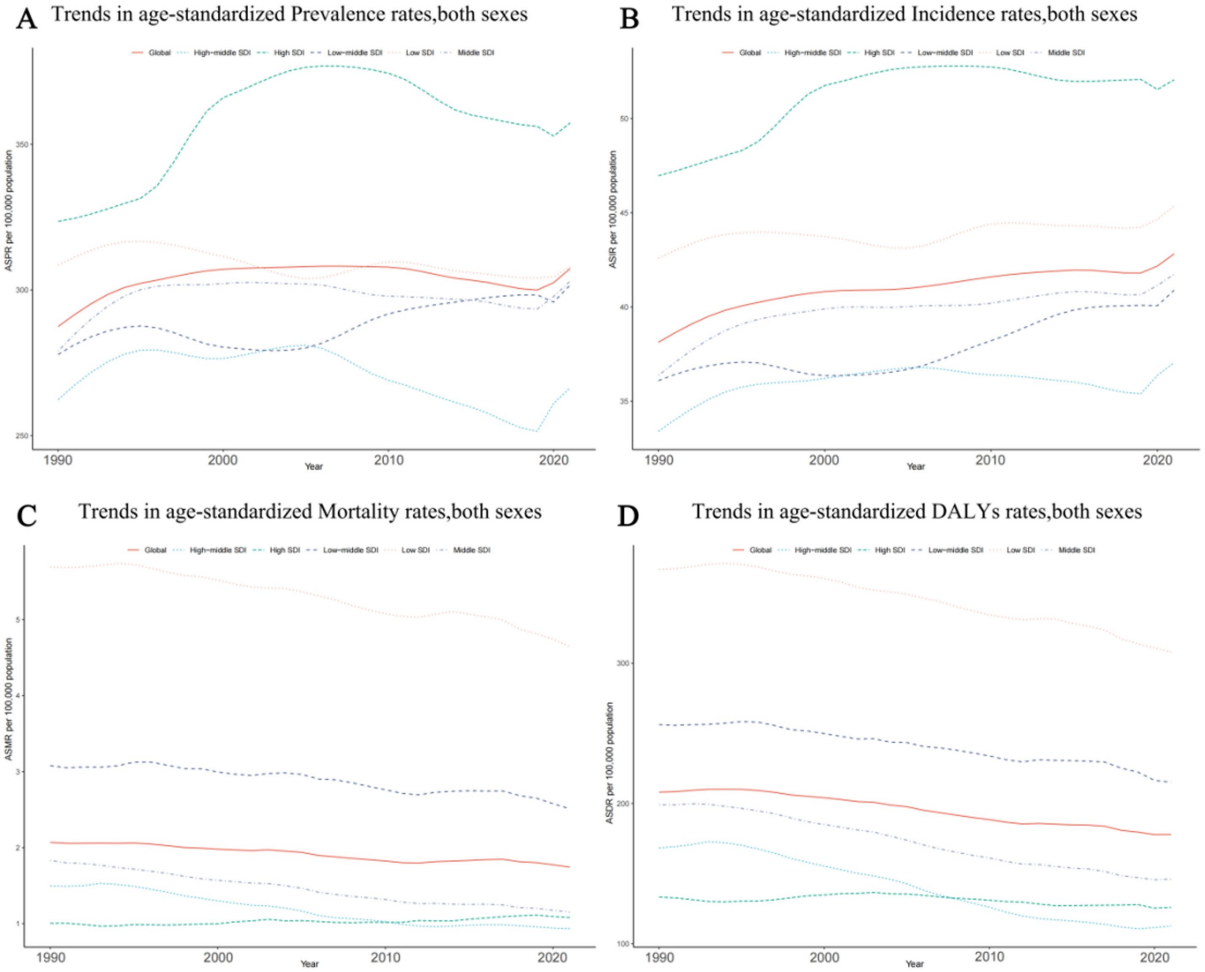
Epidemiologic trends of age-standardized rates of prevalence, incidence, deaths, and disability-adjusted life-years (DALYs) in 5 Sociodemographic Index (SDI) regions of idiopathic epilepsy from 1990 to 2021. (A) Trends in age-standardized prevalence rates, both sexes. (B) Trends in age-standardized incidence rates, both sexes. (C) Trends in age-standardized mortality rates, both sexes. (D) Trends in age-standardized DALYs rates, both sexes.

The highest rates in the epilepsy-associated ASPR occurred in high SDI region, at 357.35 per 100,000 persons (95% UI: 242.69–470.32). By contrast, high middle SDI regions demonstrated lowest ASPR at 266.45 per 100,000 persons (95% UI: 185.63–353.86). During the period from 1990 to 2021, changes in the ASPR varied, with the exception of a non-significant change of less than 0.01 (0.002) per 100,000 persons (95% CI: −0.23–0.40) in low SDI, the ASPR increase in all other SDI regions. A modest growth trend was observed in regions with high SDI, at 0.105 per 100,000 persons (95% CI: −0.116–0.313), meanwhile, low-middle SDI and middle SDI had a similar grow trend, at all around 0.09 per 100,000 persons (95% CI: −0.19–0.51) and (95% CI: −0.13–0.38) respectively (Figure 2A; Supplementary Table 3A).

#### Incidence trends

The middle SDI region had the most cases of epilepsy in 2021 with 514,418 (95% UI: 342,283–684,799). However, the highest ASIR was showed in high SDI region at 52.05 per 100,000 persons (95%UI: 34.37–70.31). Between 1990 and 2021, the ASIR has shown an increasing trend in all SDI regions. The greatest rise in the ASIR of epilepsy occurred in the middle SDI region (0.15; 95%CI: −0.08–0.45) (Figure 2B; Supplementary Table 3B).

#### Mortality trends

Among the 5 SDI regions, only the high SDI region exhibited a slight increase (0.08; 95%CI: −0.01–0.13) in epilepsy-associated mortality; Among the 4 remaining SDI regions, the high-middle SDI region and Middle SDI had the notably decrease (0.38; 95%CI: −0.45 to −0.31vs 0.37; 95%CI: −0.42 to−0.31) in ASMR; the high middle SDI region had the lowest rate of epilepsy-associated deaths in 2021(0.93; 95% UI, 0.83–1.02). On the contrary, the highest ASMR was found in the Low SDI region, reaching 4.64 per 100,000 persons (95% UI: 3.76–5.43), exceeding the global overall ASMR of 1.74 per 100,000 persons (95% UI: 1.46–1.92) (Figure 2C; Supplementary Table 3C).

#### DALYs trends

Seen from 2021, the Low SDI region still has the highest in epilepsy-associated DALYs rate at 308.08 per 100,000 persons (95% UI: 246.46–378.61), followed by the Low middle SDI region at 215.13 per 100,000 persons (95% UI: 168.21–270.45), both exceeding the global average DALYs rate. The rest of the regions are all below the global average, with the lowest being the High middle SDI region at 112.642 per 100,000 persons (95% UI: 79.87–157.06). In terms of DALYs rate changes from 1990 to 2021, all regions showed a downward trend, with the most significant decline in the High middle SDI region at −0.33 per 100,000 persons (95% CI: −0.45–0.18), while the High SDI region remained almost unchanged (−0.06; 95% CI: −0.20–0.10) (Figure 2D; Supplementary Table 3D).

### Geographic regional level

#### Prevalence trends

**Figure 3:**
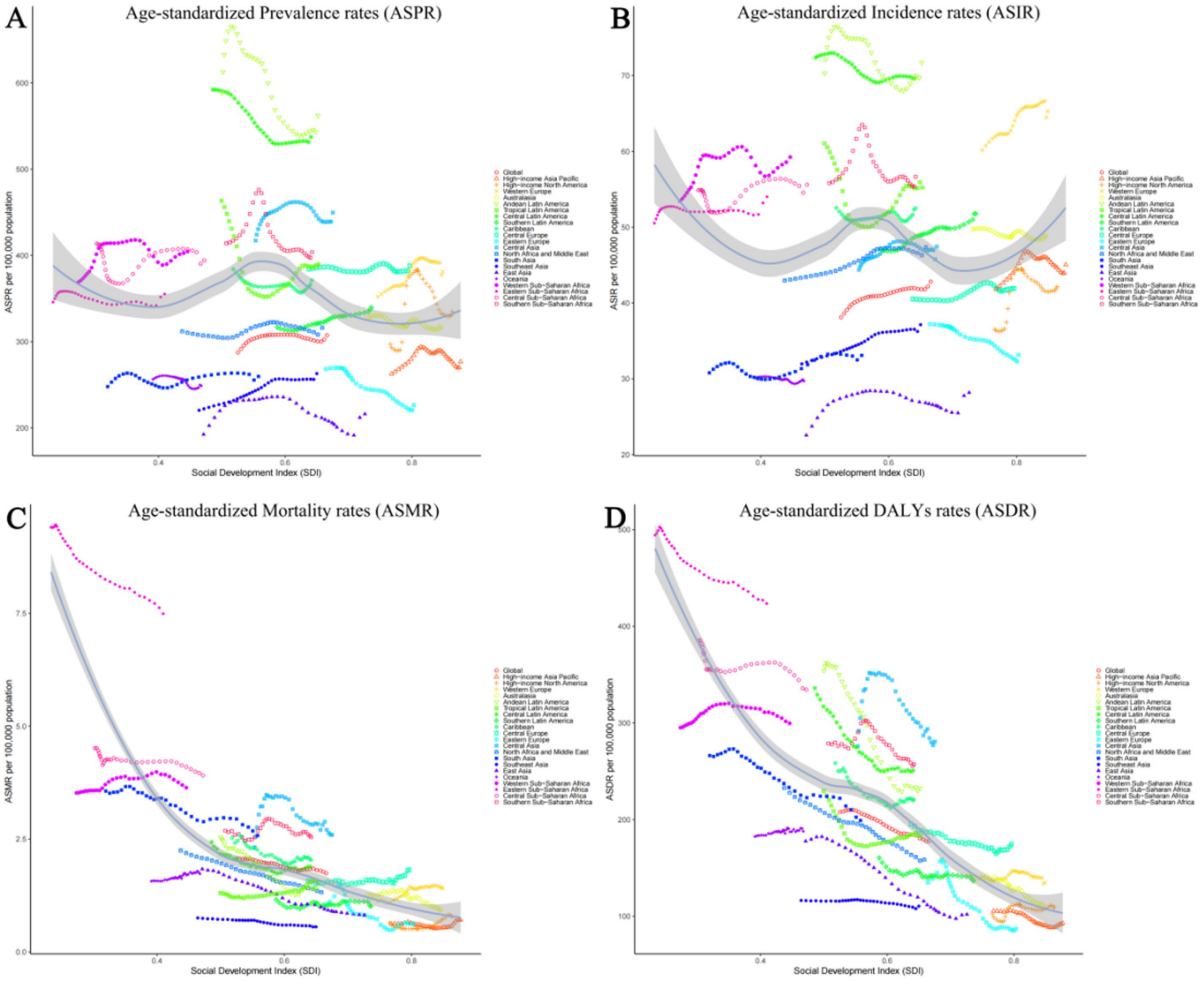
Age-standardized rates of prevalence, incidence, deaths, and disability-adjusted life-years (DALYs) in 21 GBD regions for idiopathic epilepsy from 1990 to 2021. (A) Age-standardized prevalence rates (ASPR). (B) Age-standardized incidence rates (ASIR). (C) Age-standardized mortality rates (ASMR). (D) Age-standardized DALY's rates (ASDR).

**Figure 4:**
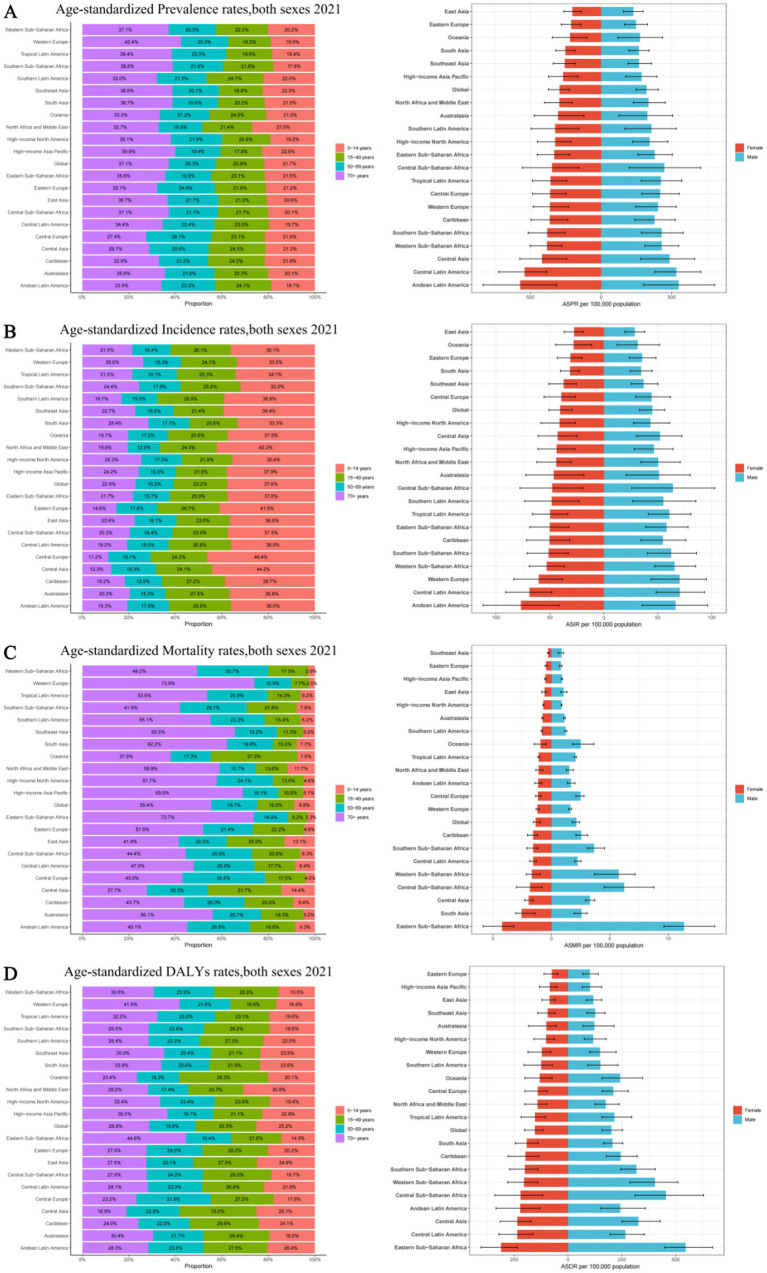
Age and sex distribution of 21 GBD regions Age-standardized rates of prevalence, incidence, deaths, and disability-adjusted life-years (DALYs) for idiopathic epilepsy in 2021. (A) Age-standardized prevalence rates, both sexes 2021. (B) Age-standardized incidence rates, both sexes 2021. (С) Age-standardized mortality rates, both sexes 2021. (D) Age-standardized DALYs rates, both sexes 2021. ASPR, Age-standardized prevalence rates; ASIR, Age-standardized incidence rates; ASMR, Age-standardized mortality rates; ASDR, Age-standardized DALYs rates.

Among 21 geographic regions, South Asia had the most cases of epilepsy in 2021 (4,707,517; 95% UI: 3,434,380–6,020,088), whereas Oceania had the fewest (33,850; 95% UI: 14,242–54,211). The ASPR of epilepsy was highest in Andean Latin America (561.91; 95% UI: 309.61–821.66). In contrast, the ASPR of epilepsy was lowest in East Asia (216.08; 95% UI: 149.91–279.23). From 1990 to 2019, Southeast Asia had the largest increase in the prevalence of epilepsy (change in ASPR, 0.20, 95% CI: −0.13–0.65), whereas the Tropical Latin America had the largest decrease (change in ASPR, 0.16; 95% CI: −0.40–0.27) (Figure 3A). Besides, there was no significant gender differences observed between these 21 regions. And the highest incidence rate was among the older adult over 70 years old in all regions (Figure 4A; Supplementary Tables 4, 5).

#### Incidence trends

The Andean Latin American region exhibits the highest ASIR of epilepsy, standing at 71.71 per 100,000 individuals (95%UI: 38.67–103.62), while Central Latin America closely follows with 69.70 per 100,000 individuals (95%UI: 48.39–92.19). In contrast, East Asia has the lowest ASIR, recording 28.18 per 100,000 individuals (95%UI: 19.18–37.38), while Oceania takes the second-lowest spot (29.71; 95%UI: 11.89–48.40). The analysis of the changes in ASIR between 1990 and 2021 shows that, except for the 6 regions of the Caribbean, Oceania, Australasia, Central Latin America, Tropical Latin America, and Eastern Europe, which showed a downward trend, all other 15 regions experienced an upward trend. Among them, East Asia had the fastest growth rate, reaching 0.25 per 100,000 individuals (95%CI: −0.10–0.73), while Eastern Europe experienced the fastest decline, with a rate of 0.11 per 100,000 individuals (95%CI: −0.32–0.15) (Figure 3B). There is no significant difference in incidence rates between men and women in different regions, but contrary to the ASPR, the incidence population is mostly concentrated in children aged 0–14, followed by young and middle-aged patients aged 14–59 (Figure 4B; Supplementary Tables 4, 5).

#### Mortality trends

Nine regions (e.g., Eastern Sub-Saharan Africa) had higher incidences of epilepsy than the global mean, whereas 12 regions (e.g., High-income North America and High-income Asia Pacific) had lower incidences than the global mean (1.74;95% UI: 1.46–1.92). The region with the highest ASMR is Eastern Sub-Saharan Africa, standing at 7.49 per 100,000 individuals (95%UI: 6.44–8.92), whereas Southeast Asia boasts the lowest figure, merely 0.56 per 100,000 individuals (95%UI: 0.40–0.66). Between 1990 and 2021, Western Europe had the most significant increase in ASMR of epilepsy (0.26; 95% CI: 0.13–0.35), while East Asia had the most significant decrease, at 0.56 (95% CI: −0.63 to −0.46) (Figure 3C). It is worth noting that there are significant gender differences in epilepsy-associated mortality rates across 21 regions, with females often having higher rates than males, and the highest mortality rate is among older adult patients over 70 years old (Figure 4C; Supplementary Tables 4, 5).

#### DALYs trends

In 2021, Eastern Sub-Saharan Africa had the highest age- standardized DALYs rate at 423.53 (95%UI: 345.14–519.67), followed by Central Sub-Saharan Africa at 334.52 (95%UI: 220.92–477.24). The lowest DALYs were observed in Eastern Europe and High-income Asia Pacific, with 87.13 (95%UI: 58.08–124.66) and 92.92 (95%UI: 59.13–144.30) respectively. From 1990 to 2021, in terms of the changes in age-standardized rates of DALYs, there was an upward trend in four regions, namely High-income North America, Oceania, Central Asia, and Western Sub-Saharan Africa. Among them, High-income North America experienced the greatest increase at 0.07 (95%UI: −0.14–0.32). All other regions showed a downward trend, with East Asia experiencing the most significant decline at −0.43 (95%CI: −0.56 to −0.25) (Figure 3D). However, there is little difference in the age-standardized rates of DALYs for epilepsy between males and females, as well as among different age groups, in various regions (Figure 4D; Supplementary Tables 4, 5).

### 204 National level

#### Prevalence trends

**Figure 5:**
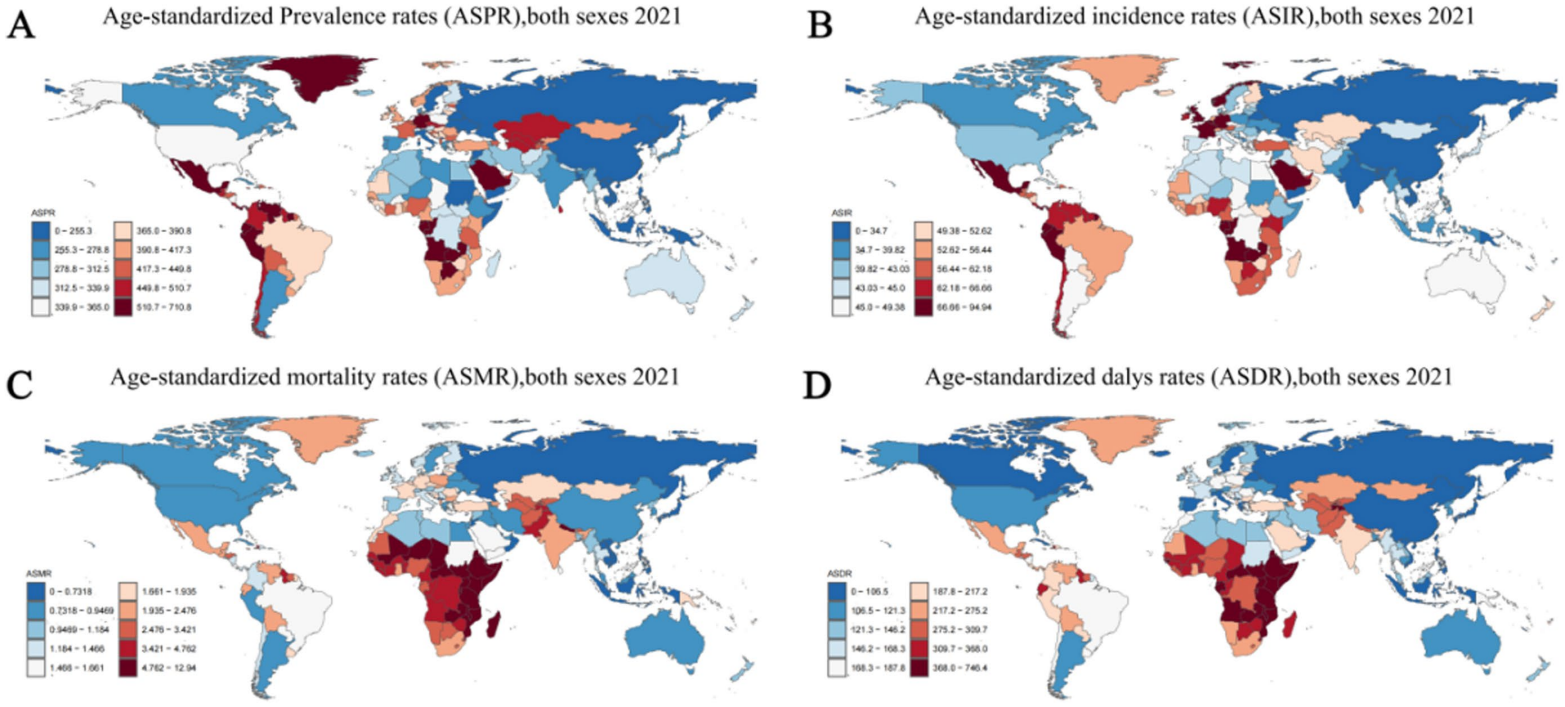
Age-standardized rates of prevalence, incidence, deaths, and disability-adjusted life-years (DALYs) of idiopathic epilepsy in 204 Countries and Territories in 2021. (A) Age-standardized prevalence rates (ASPR), both sexes 2021. (B) Age-standardized incidence rates (ASIR) both sexes 2021. (С) Age-standardized mortality rates (ASMR), both sexes 2021. (D) Age-standardized DALYs rates (ASDR), both sexes 2021.

In 2021, among 204 countries, India had the most cases of epilepsy (3,550,178; 95% UI: 2,569,547–4,559,175); Ecuador had the highest ASPR of epilepsy (710.80; 95% UI: 1,140.83–226.24). From 1990 to 2021, the ASPR showed an upward trend in 122 countries and a downward trend in 82countries.Equatorial Guinea (ASPR change, 0.754; 95% CI: 10.85–0.59) had the largest increases in epilepsy Prevalence rate; Democratic People’s Republic of Korea (ASPR change, −0.31; 95% CI: −0.83–2.04) had the largest decreases. The global ASPR of Epilepsy in 2021 was 307.38 per 100,000 persons (95% UI: 234.71–389.02); the ASPR were above the global mean in 146 countries and below the global mean in 58 countries (Figure 5A; Supplementary Table 6).

#### Incidence trends

In 2019, India had the highest number of epilepsy-associated incidence (457,420; 95% UI: 325,186–602,651), followed by China at 359,966 (95% UI: 248,027–475,661). Ecuador (94.943; 95% CI: 29.94–160.51) had the highest ASIR; Democratic People’s Republic of Korea (21.74; 95% CI: 5.91–38.69) had the lowest ASIR. From 1990 to 2021, the ASIR showed an upward trend in 143 countries and a downward trend in 61countries. Equatorial Guinea (ASIR change, 0.581; 95% CI: −0.63–10.20) had the greatest increases in the incidence rate; the Belarus (ASIR change, −0.162; 95% CI: −0.76–1.93) had the greatest decreases. The global ASIR of epilepsy in 2021 was 42.82 per 100,000 persons (95% UI: 31.24–53.72); the ASPR were above the global mean in 146countries and below the global mean in 58 countries (Figure 5B; Supplementary Table 6).

#### Mortality trends

In 2021, India had the highest number of epilepsy-associated deaths (31,176; 95% UI: 22,983–35,983), followed by China at 11,886 (95% UI: 9,815–14,767). Zambia (12.94; 95% UI: 9.47–17.08) had the highest ASIR; Viet Nam (0.08; 95% UI: 0.01–0.32) had the lowest ASIR. From 1990 to 2021, the ASMR showed an upward trend in 61 countries and a downward trend in 143 countries. Italy (ASMR change, 1.13; 95% CI: 0.74–1.38) had the greatest increases in the incidence rate; United Arab Emirates (ASMR change, −0.60; 95% CI: −0.69 to −0.44) had the greatest decreases. The global ASMR of epilepsy in 2021 was 1.74 per 100,000 persons (95% UI: 1.46–1.92); the ASPR were above the global mean in 95 countries and below the global mean in 109 countries (Figure 5C; Supplementary Table 6).

#### DALYs trends

In 2021, India had the highest number of epilepsy-associated DALYs (2,646,968; 95% UI: 2,052,364–3,304,933), followed by China at 1374719 (95% UI: 969,523–1,888,680). Zambia (746.45; 95% UI: 505.83–1031.36) had the highest ASIR; San Marino (66.98; 95% UI: 20.28–146.49) had the lowest ASIR. From 1990 to 2021, the ASDR showed an upward trend in 44 countries and a downward trend in 160 countries. Lesotho (ASDR change, 0.37; 95% CI: −0.28-1.66) had the greatest increases in the incidence rate; Turkey (ASMR change, −0.46; 95% CI: −0.71–0.00), Belarus (ASMR change, −0.45; 95% CI: −0.73–0.11) and Qatar (ASMR change, −0.45; 95% CI: −0.82–0.28) had the greatest decreases. The global ASDR of epilepsy in 2021 was 177.85 per 100,000 persons (95% UI: 137.66–225.90); the ASDR were above the global mean in 112 countries and below the global mean in 92 countries (Figure 5D; Supplementary Table 6).

### Regional disparities in epilepsy burden

In our analysis of the global burden of epilepsy, significant regional variations were observed, highlighting the heterogeneity in disease prevalence and outcomes across different geographical areas. For instance, the ASPR of epilepsy was notably higher in the South Asia region, with 4,707,517 cases reported in 2021, compared to the Oceania region, which had the lowest count at 33,850 cases. To illustrate these regional differences more vividly, we present Figures 2–5, which displays the ASPR and ASMR of epilepsy across various regions. It is evident from the figure that regions such as Andean Latin America and Central Asia exhibit higher ASPRs and ASMRs than the global average, whereas regions like East Asia and Australasia have comparatively lower rates. Furthermore, our study identified substantial regional variations in the ASIR of epilepsy. Notably, the Andean Latin American region had the highest ASIR at 71.71 per 100,000 individuals, while East Asia reported the lowest at 28.18 per 100,000 individuals.

### Risk factors for epilepsy

The GBD database identifies the risk factor for epilepsy: Behavioral risks, specifically high alcohol use, as a significant factor linked to epilepsy.

**Figure 6:**
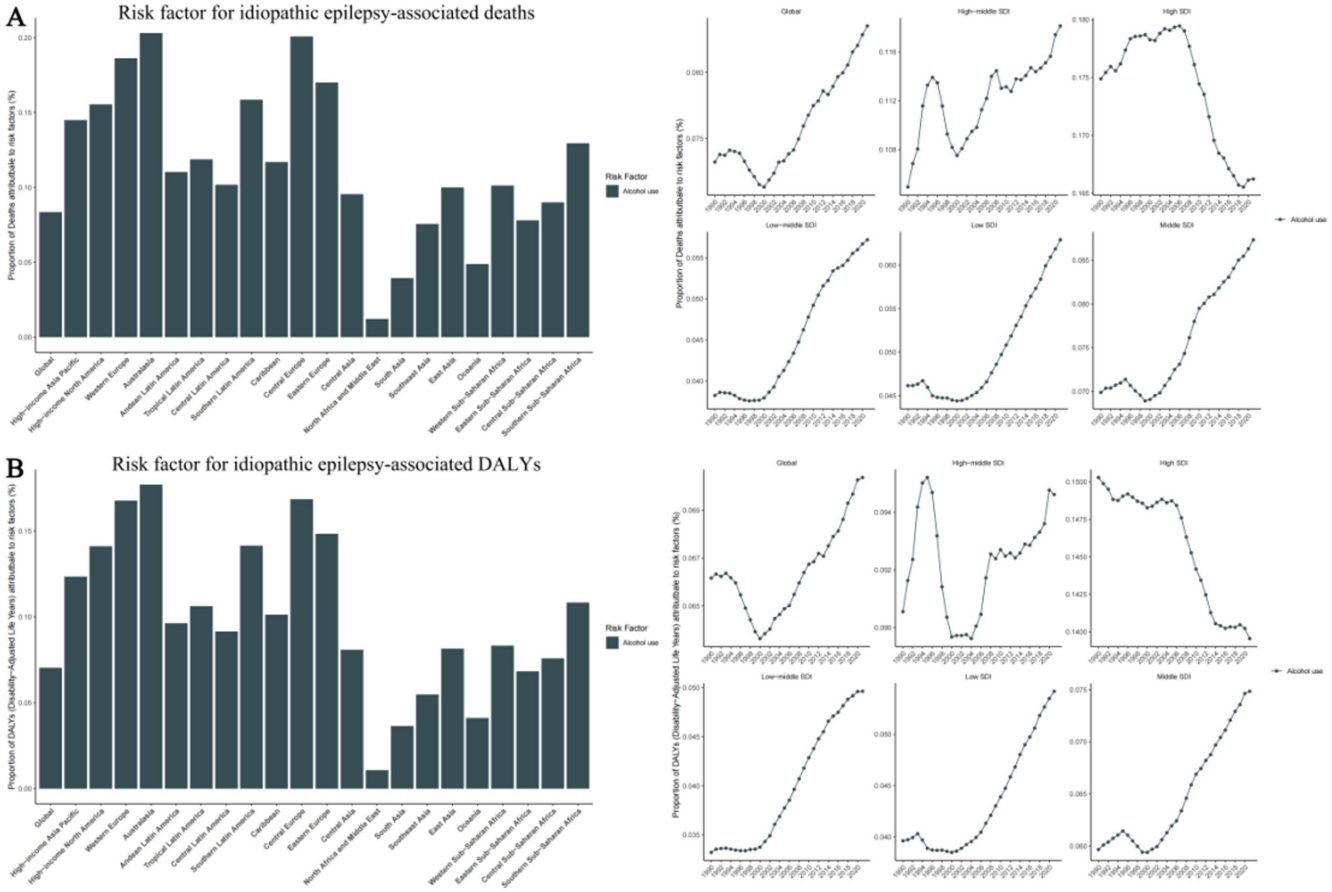
Risk factor for idiopathic epilepsy-associated deaths and disability-adjusted life-years (DALYs), with proportions in 21 GBD regions in 2021 and trends in 5 SDI Regions from 1990-2021. (A) Risk factor for idiopathic epilepsy-associated deaths. (B) Risk factor for idiopathic epilepsy-associated DALYs. SDI, Sociodemographic Index.

On a global scale, high alcohol consumption accounts for 8.4% of deaths associated with epilepsy and 7% of epilepsy-associated DALYs. More specifically, across the five SDI regions, the High SDI region sees the highest proportion of 16.6% attributed to high alcohol consumption, while the Low-middle SDI region has the lowest proportion of 5.7%. Among 21 geographical regions, Australasia and Central Europe exhibit strikingly similar proportions of 20.3 and 20.1% respectively, while Western Europe and Eastern Europe follow closely with 18.6 and 17.0%. Contrastingly, the North Africa and Middle East region has the lowest proportion of 1.2%. Notably, 5 regions -Eastern Sub-Saharan Africa, Southeast Asia, Oceania, South Asia, and North Africa have proportions below the global average (Supplementary Table 7) (Figure 6).

## Discussion

Epilepsy is a complex, multi-factorial disease influenced by various factors, characterized by abnormal electrical discharges in the brain that lead to seizures (1). The exact role of each factor in epilepsy is unknown, requires further exploration, and may vary according to geography and race. The data from this study reveal the complexity of epilepsy, particularly with regard to variations across different regions, populations, and age groups. Firstly, it was observed that the global prevalence and incidence of epilepsy have shown an upward trend from 1990 to 2021. This may reflect the impact of an aging global population, improved disease identification and reporting, and potential changes in environment and lifestyle factors. Secondly, the data indicate higher incidence rates of epilepsy among children and the older adult. Additionally, incidence and mortality rates are generally higher in males than in females. Lastly, the study’s findings reveal significant disparities between regions, which may be associated with differences in the allocation of health resources, accessibility of medical services, and variations in culture and lifestyle. In summary, the findings of this study not only uncover the global epidemiological trends of idiopathic epilepsy but also reflect the complexity of epilepsy as a disease influenced by multiple factors. These insights are of significant importance for guiding future research, improving disease management, and informing the development of public health policies.

Over the past 32 years, the numbers of epilepsy-associated deaths and DALYs have both decreased. This is supported by a systematic analysis for the GBD 2016 study, which found a significant reduction in the mortality and DALY rates in patients with epilepsy globally from 1990 to 2016 (12). DALYs is a comprehensive measure of the burden of the disease, this finding probably reflects improvements in access to treatment leading to a lower risk of death and lesser severity of the disease. The development of new therapeutic approaches and medications may have improved treatment efficacy, reducing seizure frequency and severity. Despite these improvements, the global ASPR of epilepsy increased by around 7% and the ASIR increased by almost 12%, may be linked to the aging population, where the condition is more common. Additionally, improvements in healthcare services may have increased the diagnosis rate of epilepsy and reporting could also account for higher recorded rates. Furthermore, the sedentary lifestyle and the concurrent rise in obesity may exert a synergistic effect on the incidence of epilepsy.

Our research shows that globally, the overall prevalence rate and incidence rate of epilepsy in male are higher than those in female, and the greater sexual differences in epilepsy DALY rates appeared in in areas with low SDI. Our findings provide data support for the gender research of epilepsy. Steroid hormones may serve as a potential mechanism explaining the higher incidence rate of epilepsy among males compared to females (13). Meanwhile, males have a significantly higher mortality rate than females due to their occupations and exposure to risk factors such as head trauma and alcohol consumption (14, 15). Furthermore, due to stigma and low socioeconomic status in rural areas, the rate of clinical consultation for women is often lower than that for men (14, 16). For instance, in India, women are more likely to conceal their epileptic symptoms due to sociocultural reasons (17).

As shown in Figure 1, after controlling for the effect of population, the ASIR rate of epilepsy had two peaks in 0–14, and 70+ years old, and patients in the 70+ age group had the highest DALYs rate and mortality rate. These findings are partially consistent with previous studies that show epilepsy has a bimodal distribution according to age with peaks in the youngest individuals and the older adult (18). For the 0–14 age group, the peak may be attributed to perinatal hypoxia and trauma, metabolic disorders, congenital brain malformations, and infections (13). Conditions such as perinatal and post-infectious encephalopathies, cortical dysplasia, and hippocampal sclerosis are severe forms of symptomatic epilepsies that frequently affect children (13, 19, 20). Notably, our study also reveals a peak in DALY rates among young and middle-aged individuals. In adolescents, idiopathic epilepsies account for the majority of cases, although trauma and infection play a role. Most epilepsy syndromes have complex inheritance, probably because of interacting genetic and environmental factors. Furthermore, Infants and adolescents with epilepsy have difficulty in access to diagnosis and health care. Stigmatization and poor acceptability of epilepsy impact on the quality of life and long-term outcomes of infants and adolescents with epilepsy (21). Regarding the mortality of older adult individuals over 70 years old, it is attributed to the fact that epilepsy is associated with many age- and aging-related diseases, such as Alzheimer’s disease, dementia, stroke, as well as vascular and metabolic disorders (18).

Additionally, our research and that of other scholars (11, 17) reinforce the notion of a bidirectional link between epilepsy and poverty. Crucially, our findings underscore the heavier burden of epilepsy in numerous developing regions characterized by low to lower-middle SDI scores, for example, countries in the low SDI region (e.g., Zambia, Somalia etc.) have a high epilepsy disease burden and the highest epilepsy-associated mortality rate, whereas countries in the high SDI region (e.g., San Marino, Singapore, Japan) have a low disease burden and lower epilepsy DALYs rate (Supplementary Table 7).

Lower socioeconomic status is often linked to inadequate health services, limited awareness of medical care, and infrequent outpatient clinic visits (11, 22). This trend is likely attributed to the significant economic burden of epilepsy, including the increased demand for health-care services, premature mortality, and disruption of work or education opportunities for individuals and their families (23). The high mortality rate observed in Low regions is likely due to the restricted access to medical facilities and the prevalent exposure to preventable risk factors such as drowning, head injuries, and burns, which are common in low-income countries. These factors significantly contribute to the incidence of epilepsy and its associated premature mortality (24). Nevertheless, a substantial treatment gap remains (due to insufficient financial resources, misconceptions, and stigma) that can explain the larger proportion of epilepsy and higher case fatality when related to different regions and nations (12, 22). Furthermore, certain regions may harbor unique environmental risk factors, such as exposure to infectious agents or toxins, which are posited to augment the risk of epilepsy development (25, 26).

It is noteworthy that we have found that the mortality rate of epilepsy in some Eastern European countries is higher than that in low- and middle-income countries, which is not easily explained and may reflect differences in death certification practices. However, it could also be influenced by specific regional factors such as differences in healthcare systems, access to epilepsy care, and potentially, environmental or genetic predispositions unique to these regions. The higher mortality rates in these Eastern European countries might be attributed to a lack of comprehensive epilepsy management programs, inadequate access to antiepileptic drugs, or disparities in the quality of healthcare services (27). In some Eastern European regions, historical factors such as higher levels of industrialization may have led to environmental pollution (e.g., lead exposure), increasing exposure to potential neurotoxins and possibly contributing to higher epilepsy mortality rates (28). Further research is warranted to explore these hypotheses and to understand the underlying reasons for these observed mortality differences.

Furthermore, we have found that risk factors for Epilepsy are related to behavioral habits, specifically alcohol use, which is more prevalent in high-income countries such as Australia, Western Europe, and Eastern Europe. We speculate that this may be related to the country’s dietary and cultural habits. Alcohol abuse can lead to neurological dysfunction and an increased risk of epileptic seizures. Chronic heavy drinking may result in brain structural damage, further elevating the incidence of epilepsy (29, 30). Additionally, alcohol-related accidents and injuries can cause brain trauma, which is a known risk factor for the development of epilepsy (31). Beyond alcohol use, other lifestyle factors may also play a role. Sleep deprivation (32) is a common precipitant of seizures, as it can reduce the seizure threshold. An inactive lifestyle and poor dietary habits can result in obesity, which is linked to various chronic conditions, including epilepsy (33). Moreover, smoking may impact the nervous system through the effects of nicotine (34) and other compounds in tobacco (35).

While our study has highlighted behavioral factors like alcohol use and differences in lifestyle factors as contributors to the regional burden of epilepsy, there may be other factors at play. Genetic predispositions could account for variations in epilepsy susceptibility among different populations (36, 37). Additionally, environmental exposures to toxins or infectious agents might heighten the risk of epilepsy in certain regions (26, 38). Cultural differences (39) might further impact perceptions of epilepsy and healthcare-seeking behaviors, affecting treatment outcomes. Exploring these potential factors in future research could provide a more comprehensive understanding of the determinants of regional disparities in epilepsy. Such insights are vital for developing targeted interventions to reduce the burden of epilepsy worldwide.

### Limitations

A key limitation of our study is the reliance on GBD data, which while offering a comprehensive dataset across a wide geographical range, also presents inherent limitations. The accuracy of GBD estimates is subject to the data collection and reporting capabilities of individual countries, which may introduce variability or bias into the data, particularly in regions with less robust health information systems. Secondly, the definition of epilepsy adopted in the GBD differs from the recent ILAE definition, where the GBD definition requires the occurrence of at least two unprovoked seizures, whereas the ILAE definition encompasses a single unprovoked seizure deemed to be at a high risk of recurrence (40), potentially impacting the categorization and interpretation of cases within our study. Thirdly, this study’s search was limited to the disease name ‘Idiopathic Epilepsy’ (the only one term about epilepsy) in the GBD database, the GBD dataset generally lacks granular clinical details, such as specific epilepsy syndromes, treatment regimens, or patient lifestyle factors, which limits our capacity to deeply analyze the determinants of epilepsy. Moreover, there is a scarcity of information regarding other risk factors associated with epilepsy, apart from alcohol use.

To address these and further our understanding of epilepsy, future studies should integrate multi-source data, conduct more regional research, especially in data-scarce areas, and consider a wider range of clinical, environmental, and socioeconomic factors. Additionally, gathering more clinical details, such as specific epilepsy syndromes, seizure frequencies etc., will allow for a more in-depth analysis of the factors influencing epilepsy. Furthermore, prospective cohort studies could offer valuable insights into the disease’s natural history and treatment impacts.

## Conclusion

In conclusion, our findings have significant implications for healthcare service planning and providing data support for epilepsy-related research. Over the past 32 years, despite the impact of the COVID-19 pandemic, the global mortality and DALY rates for patients with epilepsy have remained on a downward trend, which is encouraging. However, these changes vary geographically. In countries with lower SDI, the burden of epilepsy remains higher. Additionally, males tend to experience a higher burden of epilepsy compared to females, especially in less developed countries. These findings can prompt greater action in economically disadvantaged regions and the development of relevant health policies to reduce the burden of epilepsy.

## Data Availability

The original contributions presented in the study are included in the article/Supplementary material, further inquiries can be directed to the corresponding author.

## Footnotes

^1^ http://ghdx.healthdata.org/gbd-results-tool
