# Supplementary material for: A population-based analysis of the global burden of epilepsy across all age groups (1990–2021): utilizing the Global Burden of Disease 2021 data
Source: Front Neurol. 2024 Dec 12;15:1448596. doi: 10.3389/fneur.2024.1448596 (PMC11669576; doi:10.3389/fneur.2024.1448596)
Supplement: Supplementary file 14 [file Data_Sheet_1.pdf]

	Severe other endocrine, metabolic, blood, and immune disorders	0.178<br>(0.148–0.220)

Epilepsy impairment envelope

Flowchart

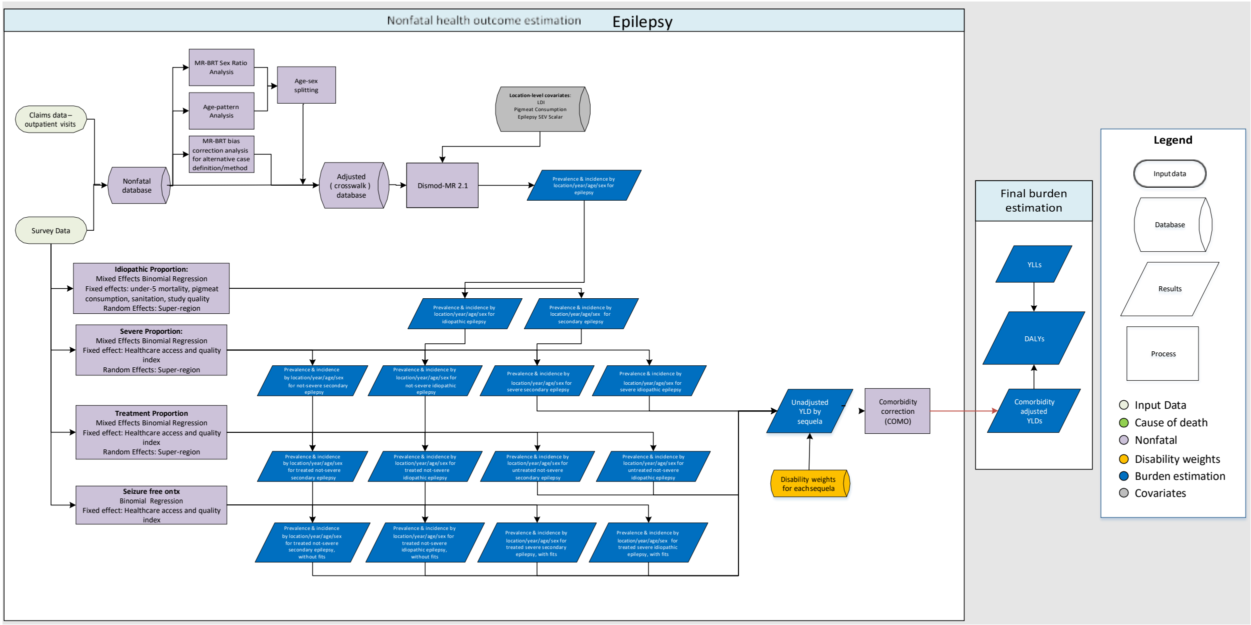

Case definition

Epilepsy is a condition characterized by recurrent epileptic seizures due to abnormal electrical activity in the brain with underlying causes including stroke, traumatic brain injury, neonatal insult to the brain, and others, including unknown origin. Since GBD 2013, we have used the following definitions from the "Guidelines for Epidemiologic Studies on Epilepsy": 1) Epilepsy: a condition characterised by recurrent (two or more) epileptic seizures, unprovoked by any immediate identified cause, and 2) "Active" epilepsy: a prevalent case of active epilepsy is defined as a person with epilepsy who has had at least one epileptic seizure in the previous five years, regardless of antiepileptic drug (AED) treatment. We also use the following ICD-10 codes for epilepsy: G40 (Neuro, epilepsy, total) and G41 (Neuro, epilepsy, status epilepticus). We define severe epilepsy as having seizures one or more times per month.

Input data and processing

Data inputs

The primary data inputs for the epilepsy modelling strategy were measurements of prevalence, incidence, remission rate, excess mortality rate, relative risk of mortality, standardised mortality ratio, or with-condition mortality rate for all epilepsy, regardless of cause, severity, or treatment status.

For GBD 2021, we conducted a systematic review covering 01/10/2016 to 01/28/2020 using the following search string:

(2016/10/01[PDAT] : 3000[PDAT]) AND ("epilepsy"[MeSH Terms] OR "epilepsy, partial, motor"[MeSH Terms] OR "epilepsy, benign neonatal"[MeSH Terms] OR "epilepsy, reflex"[MeSH Terms] OR "myoclonic epilepsy, juvenile"[MeSH Terms] OR "epilepsy, frontal lobe"[MeSH Terms] OR "epilepsy, complex partial"[MeSH Terms] OR "epilepsy, post-traumatic"[MeSH Terms] OR "epilepsy, temporal lobe"[MeSH Terms] OR "epilepsy, absence"[MeSH Terms] OR "epilepsy, tonic-clonic"[MeSH Terms] OR "epilepsies, myoclonic"[MeSH Terms] OR "epilepsies, partial"[MeSH Terms] OR epilep*[Title/Abstract]) AND (inciden*[Title/Abstract] OR prevalen*[Title/Abstract]) NOT (animals[MeSH] NOT umans[MeSH])

We included representative, population-based surveys that reported on prevalence, incidence, remission rate, excess mortality rate, relative risk of mortality, standardised mortality ratio, or with-condition mortality rate. We excluded studies with no clearly defined sample (eg, among clinic attenders or patient organisation members with non-specific or non-representative catchment area).

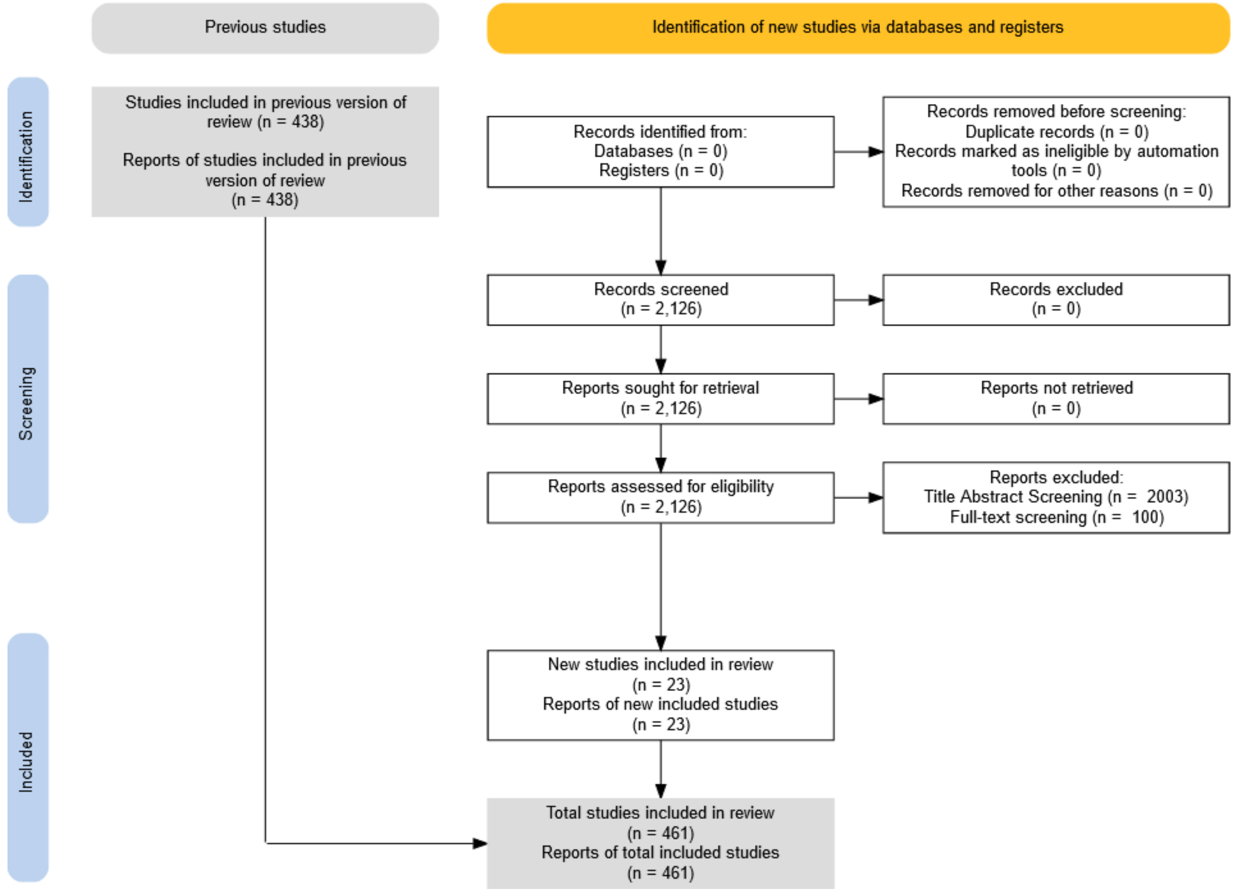

For epilepsy modelling in GBD 2021, we used the following clinical data sources: Poland claims data from 2018, and Taiwan claims data from 2016. While we have previously used USA MarketScan claims data from the years 2000, and 2010 through 2017, with the addition of the USA MarketScan claims data for

2018 we found that there was so much MarketScan claims data in comparison to the smaller population studies that MarketScan was having an unduly large impact on the model. As such we decided to drop all USA MarketScan claims data as we trust the smaller population studies more.

Additional data inputs include data on the proportion of epilepsy that is primary or idiopathic, the proportion of epilepsy that is severe (one or more fits per month), the proportion of epilepsy that is untreated (the treatment gap), and the proportion of treated epilepsy that is treated without fits (no fits reported in the preceding year).

The number of sources used for all epilepsy, and for idiopathic epilepsy specifically, are listed below:

Epilepsy impairment:

Measure	Total sources	Countries with data
All measures	491	93
Prevalence	373	87
Incidence	89	38
Remission	3	3
Other	174	56

Data processing

For GBD 2021, raw data with large age ranges were split into 5-year age groups using the age pattern generated from a Dismod-MR 2.1[1] (disease model—Bayesian meta-regression, details on this method can be found in appendix 1, section 4.5 of the citation) model with input data of only less than 25 years age range. Standard GBD sex splitting methods were used for studies with only "both"-sex datapoints. We modelled the ratio of female/male prevalence in MR-BRT (meta-regression—Bayesian, regularised, trimmed) and calculated male prevalence:

$$prev_{male} = prev_{both} * \frac{pop_{both}}{(pop_{male} + ratio * pop_{female})}$$

And then calculated female prevalence:

$$prev_{female} = ratio * prev_{male}$$

For epilepsy, the modelled female/male ratio demonstrated a higher prevalence in males and was used to proportionally split "both"-sex datapoints into male and female datapoints (as seen in the figure below).

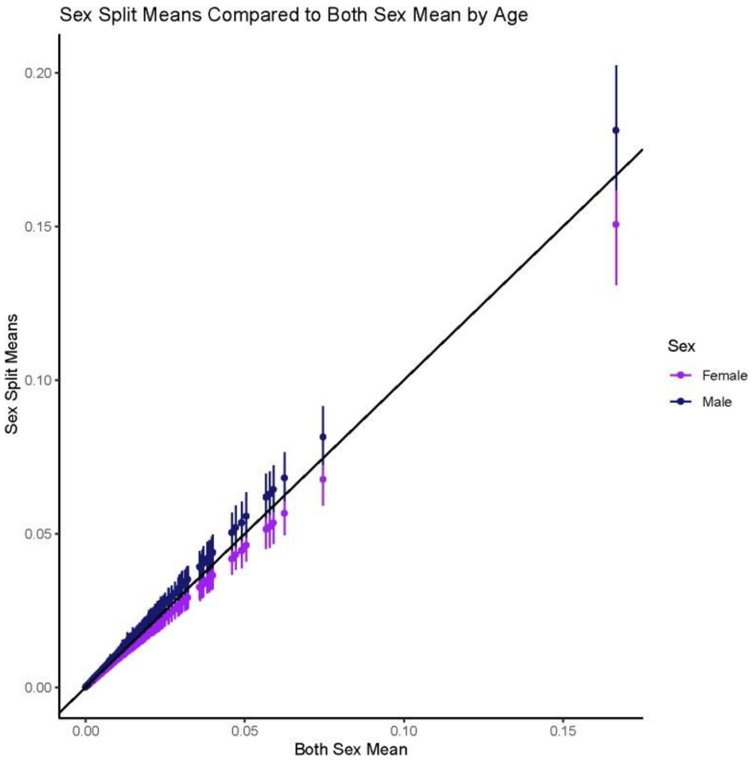

For GBD 2021, adjustment factors for all study-level covariates were determined using matched data (by year, age, sex, location) for reference and alternative case definitions in a logit ratio meta-regression. Studies that asked for lifetime recall were crosswalked to the reference definition for epilepsy (see case definition).

The table below shows adjustment factors estimated using MR-BRT.

MR-BRT crosswalk adjustment factors for epilepsy impairment envelope

Data input	Reference or alternative case definition	Gamma	Beta coefficient, log (95% UI)*	Adjustment factor**
	Ref	N/A	N/A	N/A
Recall lifetime	Alt	0.39	0.26 (–0.75 to 1.27)	1.3

**MR-BRT crosswalk adjustments can be interpreted as the factor the alternative case definition is adjusted by to reflect what it would have been had it been measured using the reference case definition. If the log/logit beta coefficient is negative, then the alternative is adjusted up to the reference. If the log/logit beta coefficient is positive, then the alternative is adjusted down to the reference.*

***The adjustment factor column is the exponentiated beta coefficient. For log beta coefficients, this is the relative rate between the two case definitions. For logit beta coefficients, this is the relative odds between the two case definitions.*

Modelling strategy

We modelled the prevalence of epilepsy in two steps: first, we created an epilepsy impairment envelope. Second, we split the envelope into primary (or idiopathic) and secondary epilepsies. Each of these was subdivided into “severe” (on average one or more fits per month) and “non-severe.” Non-severe cases were subdivided into “treated” and “untreated.” Finally, “treated” cases were divided into “treated cases with fits” (between one and 11 fits on average in the preceding year) and “treated cases without fits” (no fits reported in the preceding year).

In the first step, we used DisMod-MR 2.1 for the epilepsy impairment envelope to model a consistent fit between incidence, prevalence, remission, and fatal data.

We also included the SEV epilepsy scalar, which summarises the epilepsy risk exposure level from all epilepsy risk factors for each country, as a predictive covariate on prevalence. We included cause-specific mortality rate (CSMR) estimates from the epilepsy mortality model as input data to the DisMod-MR model. Where age-specific prevalence data were available, we calculated excess mortality rate (EMR) from prevalence and CSMR. We included the log of the lag-distributed income (LDI) as a covariate on EMR to account for lower mortality in developed countries. We included Bayesian priors on remission to account for the scarcity of remission data. We set bounds on remission from 0 to 0.25 from age 0–60 and 0 to 0.05 from age 61–100. The table below indicates the covariates used in the estimation process, as well as parameters, betas, and exponentiated betas.

Covariates. Summary of covariates used in the epilepsy impairment envelope DisMod-MR meta-regression model.

**Covariate**	**Type**	**Beta coefficient, log difference (95% UI)***	**Adjustment factor****
Log-transformed age-standardised SEV scalar: Idiopathic epilepsy	Prevalence	0.98 (0.82 to 1.14)	2.67 (2.27 to 3.14)
LDI ($ per capita)	Excess mortality rate	–0.55 (–0.97 to –0.12)	0.58 (0.38 to 0.88)

In the second step, we used mixed-effects generalised linear models (binomial family) run in GBD 2021 to predict the proportion of idiopathic epilepsy, the proportion of severe epilepsy, the proportion of treated epilepsy, and the proportion of epilepsy that is treated without fits.

Because not all the data on the proportion of idiopathic epilepsy used optimal case finding methods (using CT scans or MRIs in addition to EEGs in order to diagnose secondary epilepsy), we first ran an initial linear regression model with a covariate on study quality. We then used the beta from this model to crosswalk studies with non-optimal case finding methods to those with adequate methods. The adjusted data were then used in the regression for the proportion of epilepsy that is idiopathic, with a fixed effect on SDI as well as a random effect on super-region.

We used similar models to predict the proportion of severe epilepsy and treatment gap based on the reported proportions extracted from the systematic review. To predict the proportion of severe epilepsy and the treatment gap, we used mixed-effects models with a fixed effect on the log of Healthcare Access and Quality (HAQ) Index and a random effect on super-region.

For the regression to determine the proportion of treated epilepsy cases that had not had a fit in the last year, there was a much smaller dataset, and therefore we could not use a random effect in the model. Therefore, we used generalised linear model (binomial family) to generate predictions for the proportion of treated epilepsy that was seizure-free with a fixed effect on the log of HAQ Index.

Severity splits & disability weights
The table below illustrates the severity levels, descriptions, and disability weights associated with epilepsy. These are calculated using regressions from literature (ie, frequency of seizures).

Severity level	Lay description	Disability weights (95% CI)
severe (seizures ≥ once per month)	This person has sudden seizures one or more times each month, with violent muscle contractions and stiffness, loss of consciousness, and loss of urine or bowel control. Between seizures the person has memory loss and difficulty concentrating.	0.552 (0.375–0.71)
less severe (seizures < once per month)	This person has sudden seizures two to five times a year, with violent muscle contractions and stiffness, loss of consciousness, and loss of urine or bowel control.	0.263 (0.173–0.367)
Treated without fits	This person has a chronic disease that requires medication every day and causes some worry but minimal interference with daily activities.	0.49 (0.031–0.072)

[1] Vos T, Lim SS, Abbafati C, *et al.* Global burden of 369 diseases and injuries in 204 countries and territories, 1990–2019: a systematic analysis for the Global Burden of Disease Study 2019. *The Lancet* 2020; 396: 1204–22. doi: https://doi.org/10.1016/S0140-6736(20)30925-9

Fistula (impairment)

Flowchart
